# Cytenamide–butyric acid (1/1)

**DOI:** 10.1107/S1600536808018059

**Published:** 2008-06-19

**Authors:** Andrea Johnston, Alastair J. Florence, Francesca J. A. Fabbiani, Kenneth Shankland, Colin T. Bedford

**Affiliations:** a Solid-State Research Group, Strathclyde Institute of Pharmacy and Biomedical Sciences, The John Arbuthnott Building, University of Strathclyde, Glasgow G4 0NR, Scotland; b GZG, Department of Crystallography, University of Göttingen, D-37077 Göttingen, Germany; c ISIS Facility, Rutherford Appleton Laboratory, Chilton, Didcot, Oxon OX11 0QX, England; d Department of Chemistry, University College London, London WC1H 0AJ, England

## Abstract

Cytenamide forms a 1:1 solvate with butyric acid [systematic name: 5*H*-dibenzo[*a*,*d*]cyclo­hepta­triene-5-carboxamide–butanoic acid (1/1)], C_16_H_13_NO·C_4_H_8_O_2_. The title compound crystallizes with one mol­ecule of cytenamide and one of butyric acid in the asymmetric unit; these mol­ecules are linked by N—H⋯O and O—H⋯O hydrogen bonds to form an *R*_2_^2^(8) heterodimer motif. Pairs of adjacent motifs are further connected *via* N—H⋯O inter­actions to form a discrete centrosymmetric assembly.

## Related literature

For details on experimental methods used to obtain the title solvate, see: Davis *et al.* (1964 ▶); Florence *et al.* (2003 ▶); Florence, Johnston, Fernandes *et al.* (2006 ▶). For literature on cytenamide and related mol­ecules, see: Florence, Bedford *et al.* (2008 ▶); Cyr *et al.* (1987 ▶); Fleischman *et al.* (2003 ▶); Florence, Johnston, Price *et al.* (2006 ▶); Florence, Leech *et al.* (2006 ▶); Bandoli *et al.* (1992 ▶); Harrison *et al.* (2006 ▶); Leech *et al.* (2007 ▶); Florence, Shankland *et al.* (2008 ▶). For other related literature, see: Etter (1990 ▶) ; Desiraju & Steiner (1999 ▶).
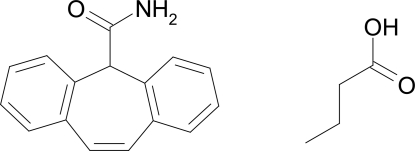


## Experimental

### 

#### Crystal data

C_16_H_13_NO·C_4_H_8_O_2_
*M* *_r_* = 323.39
Monoclinic, 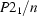

*a* = 5.9351 (2) Å
*b* = 16.3595 (5) Å
*c* = 17.6738 (4) Å
β = 98.046 (2)°
*V* = 1699.15 (9) Å^3^
*Z* = 4
Mo *K*α radiation
μ = 0.09 mm^−1^
*T* = 160 K
0.35 × 0.15 × 0.12 mm

#### Data collection

Oxford Diffraction Gemini diffractometer
Absorption correction: multi-scan (*CrysAlis RED*; Oxford Diffraction, 2007 ▶) *T*_min_ = 0.91, *T*_max_ = 0.99
18979 measured reflections
4069 independent reflections
2928 reflections with *I* > 2σ(*I*)
*R*_int_ = 0.031

#### Refinement

*R*[*F* ^2^ > 2σ(*F* ^2^)] = 0.040
*wR*(*F* ^2^) = 0.088
*S* = 0.95
4069 reflections
226 parameters
3 restraints
H atoms treated by a mixture of independent and constrained refinement
Δρ_max_ = 0.38 e Å^−3^
Δρ_min_ = −0.27 e Å^−3^

### 

Data collection: *CrysAlis CCD* (Oxford Diffraction, 2007 ▶); cell refinement: *CrysAlis RED* (Oxford Diffraction, 2007 ▶); data reduction: *CrysAlis RED* and *SORTAV* (Blessing, 1997 ▶); program(s) used to solve structure: *SIR92* (Altomare *et al.*, 1994 ▶); program(s) used to refine structure: *CRYSTALS* (Betteridge *et al.*, 2003 ▶); molecular graphics: *Mercury* (Macrae *et al.*, 2006 ▶) and *ORTEP-3* (Farrugia, 1997 ▶); software used to prepare material for publication: *publCIF* (Westrip, 2008 ▶).

## Supplementary Material

Crystal structure: contains datablocks global, I. DOI: 10.1107/S1600536808018059/gk2147sup1.cif

Structure factors: contains datablocks I. DOI: 10.1107/S1600536808018059/gk2147Isup2.hkl

Additional supplementary materials:  crystallographic information; 3D view; checkCIF report

## Figures and Tables

**Table 1:** Hydrogen-bond geometry (Å, °)

*D*—H⋯*A*	*D*—H	H⋯*A*	*D*⋯*A*	*D*—H⋯*A*
N1—H11⋯O2	0.860 (14)	2.348 (14)	2.8761 (15)	120.0 (10)
N1—H12⋯O2^i^	0.898 (13)	2.146 (13)	3.0167 (15)	163.2 (13)
O3—H311⋯O1^i^	0.879 (17)	1.698 (17)	2.5658 (13)	168.8 (16)

Symmetry code: (i) 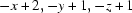.

**Fig. 1.**
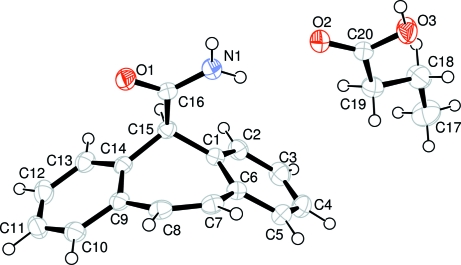
The molecular structure of CYT–butyric acid (1/1), showing 50% probablility displacement ellipsoids.

**Fig. 2.**
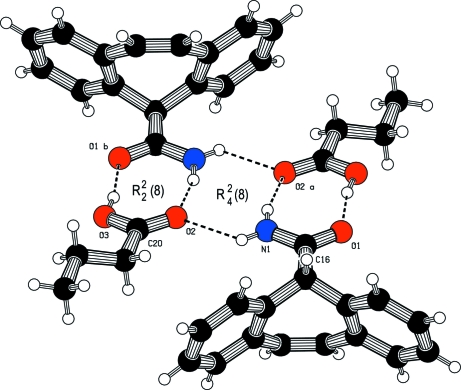
The hydrogen bonded R22(8) motifs of CYT-butyric acid joined in a centrosymmetric arrangement via an R42(8) motif. Hydrogen bonds are shown as dashed lines.

## supplementary crystallographic information

### Comment

Cytenamide (CYT) is an analogue of carbamazepine (CBZ), a dibenzazepine drug used to control seizures (Cyr *et al.*, 1987). CYT-butyric acid solvate was produced during an automated parallel crystallization study (Florence, Johnston, Fernandes *et al.*, 2006) of CYT as part of a wider investigation that couples automated parallel crystallization with crystal structure prediction methodology to investigate the basic science underlying the solid-state diversity in CBZ (Florence, Johnston, Price *et al.*, 2006; Florence, Leech *et al.*, 2006) and its closely related analogues, CYT (Florence, Bedford *et al.*, 2008), 10,11-dihydrocarbamazepine (Bandoli *et al.*, 1992; Harrison *et al.*, 2006; Leech *et al.*, 2007) and cyheptamide (Florence, Shankland *et al.*, 2008). The sample was identified as a new form using multi-sample foil transmission X-ray powder diffraction analysis (Florence *et al.*, 2003). Subsequent manual recrystallization from a saturated butyric acid solution by slow evaporation at 278 K yielded a sample suitable for single-crystal X-ray diffraction (Fig. 1).

The compound crystallizes in the monoclinic space group *P*2_1_/*n* with one CYT and one solvent molecule in the asymmetric unit. The molecules adopt a hydrogen-bonded arrangement similar to that observed in CBZ-butyric acid solvate (1/1) (Fleischman *et al.*, 2003) whereby the CYT and butyric acid molecules are connected *via* N—H···O and O—H···O hydrogen bonds to form an *R*_2_^2^(8) dimer motif (Etter, 1990). Adjacent dimers are linked *via* a third contact (N1—H1···O2; Fig 2) to form an *R*_4_^2^(8) centrosymmetric double motif arrangement. The O1···O3 distance of 2.566 (1) Å lies within the expected range for strong hydrogen bonds (2.5 - 3.2 Å; Desiraju and Steiner, 1999).

CYT-butyric acid solvate structure reported here is essentially isostructural with both CBZ-formic acid and CBZ-acetic acid solvates (Fleischman *et al.*, 2003).

### Experimental

A sample of cytenamide was synthesized according to a modification of the published method (Davis *et al.*, 1964). A single-crystal sample of cytenamide-butyric acid was grown form a saturated butyric acid solution by isothermal solvent evaporation at 278 K.

### Refinement

H-atoms were found on a difference Fourier map and were initially refined with soft restraints on the bond lengths and angles to regularize their geometry and *U*_iso_(H) (in the range 1.2–1.5 times *U*_eq_ of the parent atom), after which the positions were refined with riding constraints. The positions of H-atoms involved in H-bonding were refined subject to distance restraints.

### Crystal data

**Table:**

C_16_H_13_NO·C_4_H_8_O_2_	*F*_000_ = 688
*M**_r_* = 323.39	*D*_x_ = 1.264 Mg m^−^^3^
Monoclinic, *P*2_1_/*n*	Melting point: 216.2 K
Hall symbol: -P 2yn	Mo *K*α radiation λ = 0.71073 Å
*a* = 5.9351 (2) Å	Cell parameters from 6486 reflections
*b* = 16.3595 (5) Å	θ = 3–29º
*c* = 17.6738 (4) Å	µ = 0.09 mm^−^^1^
β = 98.046 (2)º	*T* = 160 K
*V* = 1699.15 (9) Å^3^	Block, colourless
*Z* = 4	0.35 × 0.15 × 0.12 mm

### Data collection

**Table:**

Oxford Diffraction Gemini diffractometer	4069 independent reflections
Radiation source: Enhance (Mo) X-ray Source	2928 reflections with *I* > 2σ(*I*)
Monochromator: graphite	*R*_int_ = 0.031
Detector resolution: 15.9745 pixels mm^-1^	θ_max_ = 28.7º
*T* = 160 K	θ_min_ = 2.6º
ω scans	*h* = −7→7
Absorption correction: multi-scan(CrysAlis RED; Oxford Diffraction, 2007)	*k* = 0→21
*T*_min_ = 0.91, *T*_max_ = 0.99	*l* = 0→23
18979 measured reflections	

### Refinement

**Table:**

Refinement on *F*^2^	Primary atom site location: structure-invariant direct methods
Least-squares matrix: full	Hydrogen site location: geom+difmap
*R*[*F*^2^ > 2σ(*F*^2^)] = 0.040	H atoms treated by a mixture of independent and constrained refinement
*wR*(*F*^2^) = 0.088	Method = Modified Sheldrick *w* = 1/[σ^2^(*F*^2^) + (0.03*P*)^2^ + 0.5*P*], where *P* = [max(*F*_o_^2^,0) + 2*F*_c_^2^]/3
*S* = 0.95	(Δ/σ)_max_ = 0.001
4069 reflections	Δρ_max_ = 0.38 e Å^−^^3^
226 parameters	Δρ_min_ = −0.27 e Å^−^^3^
3 restraints	Extinction correction: none

### Fractional atomic coordinates and isotropic or equivalent isotropic displacement parameters (Å^2^)

**Table:**

	*x*	*y*	*z*	*U*_iso_*/*U*_eq_	
C1	0.3604 (2)	0.32253 (7)	0.41898 (7)	0.0261	
C2	0.1718 (2)	0.36489 (8)	0.38294 (7)	0.0323	
C3	0.1256 (2)	0.36854 (9)	0.30423 (8)	0.0391	
C4	0.2687 (2)	0.33058 (9)	0.25996 (7)	0.0392	
C5	0.4591 (2)	0.28990 (8)	0.29445 (7)	0.0344	
C6	0.5061 (2)	0.28359 (7)	0.37441 (7)	0.0279	
C7	0.6996 (2)	0.23388 (8)	0.40716 (7)	0.0304	
C8	0.7115 (2)	0.18488 (8)	0.46816 (7)	0.0301	
C9	0.5383 (2)	0.17092 (8)	0.51782 (6)	0.0269	
C10	0.5251 (2)	0.09343 (8)	0.55037 (7)	0.0341	
C11	0.3603 (2)	0.07491 (9)	0.59538 (8)	0.0398	
C12	0.2069 (2)	0.13407 (9)	0.61005 (8)	0.0389	
C13	0.2205 (2)	0.21186 (8)	0.58012 (7)	0.0321	
C14	0.3844 (2)	0.23123 (7)	0.53434 (6)	0.0258	
C15	0.4034 (2)	0.31760 (7)	0.50530 (6)	0.0257	
C16	0.6242 (2)	0.35710 (7)	0.54306 (7)	0.0276	
C17	0.3158 (3)	0.57421 (11)	0.16558 (9)	0.0568	
C18	0.5184 (3)	0.60108 (10)	0.22080 (8)	0.0455	
C19	0.5957 (2)	0.53835 (9)	0.28103 (8)	0.0371	
C20	0.8040 (2)	0.56040 (8)	0.33497 (7)	0.0295	
O1	0.70946 (16)	0.33413 (6)	0.60746 (5)	0.0369	
O2	0.84351 (15)	0.53413 (6)	0.39986 (5)	0.0346	
N1	0.7091 (2)	0.41860 (7)	0.50777 (6)	0.0335	
O3	0.94149 (17)	0.61035 (6)	0.30567 (5)	0.0435	
H151	0.2828	0.3488	0.5249	0.0293*	
H21	0.0713	0.3916	0.4145	0.0377*	
H81	0.8456	0.1498	0.4786	0.0333*	
H71	0.8293	0.2332	0.3790	0.0372*	
H191	0.4766	0.5266	0.3115	0.0486*	
H192	0.6295	0.4889	0.2564	0.0473*	
H131	0.1155	0.2536	0.5914	0.0383*	
H101	0.6291	0.0526	0.5389	0.0392*	
H41	0.2338	0.3317	0.2057	0.0465*	
H121	0.0935	0.1222	0.6419	0.0464*	
H51	0.5637	0.2636	0.2641	0.0395*	
H111	0.3507	0.0214	0.6150	0.0470*	
H182	0.4770	0.6515	0.2459	0.0609*	
H181	0.6503	0.6120	0.1946	0.0611*	
H31	−0.0099	0.3971	0.2794	0.0463*	
H172	0.2712	0.6162	0.1279	0.0868*	
H173	0.1858	0.5625	0.1933	0.0867*	
H171	0.3504	0.5240	0.1402	0.0875*	
H12	0.834 (2)	0.4432 (9)	0.5320 (8)	0.0446*	
H11	0.652 (2)	0.4352 (9)	0.4630 (8)	0.0433*	
H311	1.058 (3)	0.6243 (11)	0.3395 (9)	0.0689*	

### Atomic displacement parameters (Å^2^)

**Table:**

	*U*^11^	*U*^22^	*U*^33^	*U*^12^	*U*^13^	*U*^23^
C1	0.0283 (6)	0.0234 (6)	0.0265 (6)	−0.0013 (5)	0.0032 (5)	−0.0008 (5)
C2	0.0315 (7)	0.0305 (7)	0.0348 (7)	0.0030 (5)	0.0040 (5)	0.0027 (5)
C3	0.0366 (8)	0.0396 (8)	0.0383 (7)	0.0032 (6)	−0.0041 (6)	0.0075 (6)
C4	0.0476 (8)	0.0422 (8)	0.0259 (6)	−0.0032 (7)	−0.0010 (6)	0.0030 (6)
C5	0.0414 (8)	0.0345 (8)	0.0279 (6)	−0.0020 (6)	0.0075 (6)	−0.0025 (5)
C6	0.0297 (6)	0.0258 (7)	0.0279 (6)	−0.0019 (5)	0.0031 (5)	−0.0021 (5)
C7	0.0287 (6)	0.0318 (7)	0.0315 (6)	0.0018 (5)	0.0065 (5)	−0.0068 (5)
C8	0.0270 (6)	0.0295 (7)	0.0321 (6)	0.0056 (5)	−0.0016 (5)	−0.0067 (5)
C9	0.0273 (6)	0.0284 (7)	0.0227 (6)	−0.0004 (5)	−0.0051 (5)	−0.0026 (5)
C10	0.0388 (7)	0.0291 (7)	0.0313 (6)	0.0021 (6)	−0.0057 (6)	−0.0015 (5)
C11	0.0507 (9)	0.0310 (8)	0.0347 (7)	−0.0078 (6)	−0.0051 (6)	0.0073 (6)
C12	0.0384 (8)	0.0448 (9)	0.0327 (7)	−0.0111 (7)	0.0021 (6)	0.0063 (6)
C13	0.0298 (7)	0.0379 (8)	0.0275 (6)	−0.0022 (6)	0.0010 (5)	−0.0008 (5)
C14	0.0264 (6)	0.0277 (7)	0.0215 (5)	−0.0016 (5)	−0.0027 (5)	−0.0024 (5)
C15	0.0270 (6)	0.0256 (7)	0.0252 (6)	0.0034 (5)	0.0055 (5)	−0.0028 (5)
C16	0.0334 (7)	0.0245 (6)	0.0252 (6)	0.0009 (5)	0.0059 (5)	−0.0041 (5)
C17	0.0518 (10)	0.0666 (12)	0.0470 (9)	0.0033 (8)	−0.0109 (7)	−0.0040 (8)
C18	0.0467 (9)	0.0430 (9)	0.0435 (8)	−0.0033 (7)	−0.0052 (7)	0.0042 (7)
C19	0.0398 (8)	0.0347 (8)	0.0361 (7)	−0.0062 (6)	0.0026 (6)	−0.0017 (6)
C20	0.0366 (7)	0.0246 (7)	0.0279 (6)	−0.0004 (5)	0.0066 (5)	−0.0014 (5)
O2	0.0421 (5)	0.0326 (5)	0.0290 (5)	−0.0029 (4)	0.0045 (4)	0.0035 (4)
N1	0.0402 (7)	0.0304 (6)	0.0291 (5)	−0.0056 (5)	0.0024 (5)	0.0018 (5)
O3	0.0457 (6)	0.0529 (7)	0.0297 (5)	−0.0205 (5)	−0.0027 (4)	0.0077 (4)
O1	0.0441 (5)	0.0373 (5)	0.0271 (4)	−0.0124 (4)	−0.0027 (4)	0.0021 (4)

### Geometric parameters (Å, °)

**Table:**

C1—C2	1.3924 (17)	C12—H121	0.956
C1—C6	1.4018 (17)	C13—C14	1.3860 (17)
C1—C15	1.5133 (15)	C13—H131	0.965
C2—C3	1.3811 (18)	C14—C15	1.5129 (17)
C2—H21	0.975	C15—C16	1.5282 (17)
C3—C4	1.380 (2)	C15—H151	0.981
C3—H31	0.980	C16—N1	1.3204 (16)
C4—C5	1.378 (2)	C16—O1	1.2376 (14)
C4—H41	0.952	C17—C18	1.504 (2)
C5—C6	1.4054 (17)	C17—H172	0.968
C5—H51	0.976	C17—H173	0.988
C6—C7	1.4591 (17)	C17—H171	0.971
C7—C8	1.3373 (18)	C18—C19	1.5034 (19)
C7—H71	0.974	C18—H182	0.984
C8—C9	1.4601 (18)	C18—H181	0.980
C8—H81	0.977	C19—C20	1.4956 (18)
C9—C10	1.3988 (18)	C19—H191	0.967
C9—C14	1.4026 (17)	C19—H192	0.954
C10—C11	1.3778 (19)	C20—O2	1.2165 (14)
C10—H101	0.950	C20—O3	1.3114 (15)
C11—C12	1.378 (2)	N1—H12	0.899 (14)
C11—H111	0.946	N1—H11	0.860 (14)
C12—C13	1.3848 (19)	O3—H311	0.880 (14)
			
C2—C1—C6	119.25 (11)	C14—C13—H131	119.0
C2—C1—C15	119.95 (11)	C9—C14—C13	119.42 (12)
C6—C1—C15	120.79 (10)	C9—C14—C15	120.31 (11)
C1—C2—C3	121.06 (12)	C13—C14—C15	120.22 (11)
C1—C2—H21	118.6	C1—C15—C14	112.44 (10)
C3—C2—H21	120.4	C1—C15—C16	115.54 (10)
C2—C3—C4	120.03 (13)	C14—C15—C16	110.29 (10)
C2—C3—H31	120.5	C1—C15—H151	107.5
C4—C3—H31	119.5	C14—C15—H151	105.8
C3—C4—C5	119.87 (12)	C16—C15—H151	104.4
C3—C4—H41	119.9	C15—C16—N1	118.47 (11)
C5—C4—H41	120.2	C15—C16—O1	119.24 (11)
C4—C5—C6	121.01 (12)	N1—C16—O1	122.14 (12)
C4—C5—H51	121.0	C18—C17—H172	110.8
C6—C5—H51	118.0	C18—C17—H173	110.1
C5—C6—C1	118.72 (11)	H172—C17—H173	108.7
C5—C6—C7	118.30 (11)	C18—C17—H171	110.2
C1—C6—C7	122.92 (11)	H172—C17—H171	109.7
C6—C7—C8	127.14 (12)	H173—C17—H171	107.2
C6—C7—H71	116.0	C17—C18—C19	113.39 (13)
C8—C7—H71	116.7	C17—C18—H182	108.1
C7—C8—C9	128.04 (12)	C19—C18—H182	108.9
C7—C8—H81	117.0	C17—C18—H181	111.4
C9—C8—H81	114.7	C19—C18—H181	105.9
C8—C9—C10	118.27 (11)	H182—C18—H181	109.0
C8—C9—C14	123.29 (11)	C18—C19—C20	115.46 (11)
C10—C9—C14	118.45 (11)	C18—C19—H191	110.9
C9—C10—C11	121.46 (13)	C20—C19—H191	107.3
C9—C10—H101	118.2	C18—C19—H192	108.6
C11—C10—H101	120.2	C20—C19—H192	106.7
C10—C11—C12	119.67 (13)	H191—C19—H192	107.6
C10—C11—H111	120.0	C19—C20—O2	123.28 (12)
C12—C11—H111	120.3	C19—C20—O3	113.77 (11)
C11—C12—C13	119.87 (13)	O2—C20—O3	122.95 (12)
C11—C12—H121	120.4	C16—N1—H12	117.5 (10)
C13—C12—H121	119.7	C16—N1—H11	123.2 (10)
C12—C13—C14	121.09 (13)	H12—N1—H11	119.3 (14)
C12—C13—H131	119.9	C20—O3—H311	111.5 (12)
			
C6—C1—C2—C3	0.48 (19)	C14—C9—C10—C11	2.43 (18)
C15—C1—C2—C3	−178.34 (12)	C8—C9—C14—C13	177.89 (11)
C2—C1—C6—C5	1.17 (17)	C8—C9—C14—C15	−4.73 (17)
C2—C1—C6—C7	−176.25 (11)	C10—C9—C14—C13	−1.96 (17)
C15—C1—C6—C5	180.00 (12)	C10—C9—C14—C15	175.42 (10)
C15—C1—C6—C7	2.57 (18)	C9—C10—C11—C12	−1.2 (2)
C2—C1—C15—C14	114.81 (12)	C10—C11—C12—C13	−0.6 (2)
C2—C1—C15—C16	−117.37 (12)	C11—C12—C13—C14	1.0 (2)
C6—C1—C15—C14	−63.99 (14)	C12—C13—C14—C9	0.28 (18)
C6—C1—C15—C16	63.83 (14)	C12—C13—C14—C15	−177.10 (11)
C1—C2—C3—C4	−0.7 (2)	C9—C14—C15—C1	64.61 (14)
C2—C3—C4—C5	−0.8 (2)	C9—C14—C15—C16	−65.93 (13)
C3—C4—C5—C6	2.5 (2)	C13—C14—C15—C1	−118.03 (12)
C4—C5—C6—C1	−2.67 (18)	C13—C14—C15—C16	111.43 (12)
C4—C5—C6—C7	174.87 (12)	C1—C15—C16—O1	−156.56 (11)
C1—C6—C7—C8	36.0 (2)	C1—C15—C16—N1	27.97 (15)
C5—C6—C7—C8	−141.46 (14)	C14—C15—C16—O1	−27.68 (15)
C6—C7—C8—C9	−1.9 (2)	C14—C15—C16—N1	156.85 (11)
C7—C8—C9—C10	147.22 (13)	C17—C18—C19—C20	177.01 (13)
C7—C8—C9—C14	−32.6 (2)	C18—C19—C20—O2	152.97 (13)
C8—C9—C10—C11	−177.43 (12)	C18—C19—C20—O3	−27.50 (17)

### Hydrogen-bond geometry (Å, °)

**Table:**

*D*—H···*A*	*D*—H	H···*A*	*D*···*A*	*D*—H···*A*
N1—H11···O2	0.860 (14)	2.348 (14)	2.8761 (15)	120.0 (10)
N1—H12···O2^i^	0.898 (13)	2.146 (13)	3.0167 (15)	163.2 (13)
O3—H311···O1^i^	0.879 (17)	1.698 (17)	2.5658 (13)	168.8 (16)

Symmetry codes: (i) −*x*+2, −*y*+1, −*z*+1.
